# New digital confocal laser microscopy may boost real-time evaluation of endoscopic ultrasound-guided fine-needle biopsy (EUS-FNB) from solid pancreatic lesions: Data from an international multicenter study

**DOI:** 10.1016/j.ebiom.2022.104377

**Published:** 2022-11-24

**Authors:** Isabel Amendoeira, Paolo Giorgio Arcidiacono, Jessica Barizzi, Arrigo Capitanio, Miriam Cuatrecasas, Francesco Maria Di Matteo, Claudio Doglioni, Noriyoshi Fukushima, Franco Fulciniti, Angels Ginès, Marc Giovannini, Li Zaibo, Joanne Lopes, Giovanni Lujan, Alice Parisi, Flora Poizat, Luca Reggiani Bonetti, Serena Stigliano, Chiara Taffon, Martina Verri, Anna Crescenzi

**Affiliations:** a Serviço de Anatomia Patológica, Centro Hospitalar Universitário de S. João (CHUSJ) and Ipatimup, Porto, Portugal; b Bilio-Pancreatic and Endosonography Unit, IRCCS Ospedale San Raffaele di Milano, Italy; c Istituto Cantonale di Patologia, Locarno, Switzerland; d Department of Clinical Pathology, and Department of Clinical and Experimental Medicine, Linköping University, Linköping, Sweden; e Pathology Department, Center of Biomedical Diagnosis (CDB), Hospital Clínic, University of Barcelona, IDIBAPS, Spain; f Endoscopic Unit, Fondazione Policlinico Universitario Campus Bio-Medico, Rome, Italy; g Department of Pathology, San Raffaele Scientific Institute, Milan, Italy; h Department of Pathology, Jichi Medical University, Shimotsuke, Tochigi, 329-0498, Japan; i Endoscopy Unit. ICMDM, Hospital Clínic. Barcelona, Spain; j Chief of Gastroenterology Department at Paoli-Calmettes Institute, France; k Department of Pathology, The Ohio State University Wexner Medical Center, Columbus, OH, USA; l Department of Pathology, Azienda Ospedaliera Universitaria Integrata di Verona, Verona Italy; m Department of Biopathology, Institut Paoli-Calmettes, Marseille, France; n Department of Pathology, University of Modena and Reggio Emilia, Modena, Italy; o Unit of Pathology of Endocrine Organs and Neuro-muscolar Pathology, Fondazione Policlinico Universitario Campus Bio-Medico, Rome, Italy

**Keywords:** EUS-FNB, Digital pathology, Multicenter study, Pancreatic cancer, Ex-vivo fluorescence confocal laser microscopy, Inter-observer agreement

## Abstract

**Background:**

Pancreatic cancer is an aggressive malignancy and a leading cause of cancer death worldwide; its lethality is partly linked to the difficulty of early diagnosis. Modern devices for endoscopic ultrasound-guided fine-needle biopsy (EUS-FNB) were recently developed to improve targeting and sampling of small lesions, but innovative technologies for microscopic assessment are still lacking. Ex vivo fluorescence confocal laser microscopy (FCM) is a new digital tool for real-time microscopic assessment of fresh unfixed biological specimens, avoiding conventional histological slide preparation and potentially being highly appealing for EUS-FNB specimens.

**Methods:**

This study evaluated the possible role of FCM for immediate evaluation of pancreatic specimens from EUS-FNB. It involved comparison of the interobserver agreement between the new method and standard histological analysis during international multicenter sharing of digital images. Digital images from 25 cases of EUS-FNB obtained with real-time FCM technology and 25 paired digital whole-slide images from permanent conventional paraffin sections were observed by 10 pathologists from different Institutions in Europe, Japan, and the United States, in a blinded manner. The study evaluated 500 observations regarding adequacy, morphological clues, diagnostic categories, and final diagnosis.

**Findings:**

Statistical analysis showed substantial equivalence in the interobserver agreement among pathologists using the two techniques. There was also good inter-test agreement in determining sample adequacy and when assigning a diagnostic category. Among morphological features, nuclear enlargement was the most reproducible clue, with very good inter-test agreement.

**Interpretation:**

Findings in this study are from international multicenter digital sharing and are published here for the first time. Considering the advantages of FCM digital diagnostics in terms of reduced time and unaltered sample maintenance, the ex vivo confocal laser microscopy may effectively improve traditional EUS-FNB diagnostics, with significant implications for planning modern diagnostic workflow for pancreatic tumors.

**Funding:**

This study was not supported by any funding source.

**Research in context**

**Evidence before this study**

Pancreatic cancer is a highly lethal cancer with very few therapeutic options. Early diagnosis is required to maximize the likelihood of survival and endoscopic sampling is the standard procedure for diagnosing pancreatic tumors. Modern devices for endoscopic ultrasound-guided fine-needle biopsy (EUS-FNB) were recently developed to improve diagnostic accuracy by simultaneously obtaining cytological aspirates and histologic core samples. However, innovative technologies are still required for microscopic evaluation of these samples and specimens are sometimes inadequate for diagnostic evaluation or integrative analysis. Ex vivo fluorescence confocal laser microscopy (FCM) is a new digital tool for real-time microscopic assessment of fresh unfixed biological specimens. The advantage provided by this approach involves the production of digital histological images without the need for conventional slide preparation, which appears particularly appealing for EUS-FNB specimens. We have carefully considered all of the scientific literature on modern EUS-FNB and the applications of FCM technology. Given that both of these fields are extremely recently established, we surveyed literature from the last 8 years using the most popular medical databases (PubMed, MEDLINE, the Cochrane Library, Web of Science). The search was limited to English language publications. The study design consisted of an international multicenter web-based interobserver agreement evaluation of pancreatic EUS-FNB on FCM digital images and paired digital slides of conventional paraffin sections.

**Added value of this study**

This study evaluated the validity of digital FCM through comparing the interobserver agreement between the new method and standard histological analysis. The obtained data have not previously been published and they show substantial equivalence in the diagnostic performance of the two techniques. Considering the advantages of instant digital diagnostics in terms of short time procedures and intact samples preservation, the ex vivo confocal laser microscopy may effectively improve traditional microscopic diagnostic methods in fine-needle sampling of pancreatic lesions.

**Implications of all the available evidence**

This leading multicenter study on the use of FCM for pancreatic EUS-FNB indicates its value as a diagnostic tool capable of improving assessment and real-time diagnosis of pancreatic solid lesions, thereby reducing unnecessary delays in patient management. The obtained results may have significant implications for planning modern diagnostic workflow for pancreatic tumors.

## Introduction

Pancreatic cancer is an aggressive and lethal malignancy and a leading cause of cancer death worldwide.^1^ Given the difficulty in achieving diagnosis before an advanced disease stage, the 5-year survival rate is as low as 9%,^2^ so improvements in both prevention and early diagnosis of this disease are required. Endoscopic ultrasound-guided fine-needle biopsy (EUS-FNA/FNB) has become the standard procedure for sampling pancreatic solid lesions. Several technological solutions have recently been developed to improve both lesions targeting and the collection of cyto-histological material for diagnosis. Modern ultrasound devices enable the targeting of deeply located lesions, while newly developed needles allow good quality material to be obtained even using small needle gauges.^3^ However, these technological advancements have not been accompanied by progress in the preparation, adequacy assessment, microscopic evaluation, and diagnosis of obtained biological specimens. The accuracy of cytopathologic diagnosis of pancreatic solid lesions ranges from 78% to 95%.^4^ Recent meta-analysis reported sensitivity of 0.84 [95% confidence interval (CI) 0.82–0.87] and specificity of 0.98 (95% CI, 0.93–1.00) for EUS-FNB in solid pancreatic lesions with good diagnostic accuracy (0.90).^5^ Rapid on-site evaluation (ROSE) by an expert cytopathologist is recommended because of its impact on the EUS/FNA-FNB adequacy rate, but is not always available.^6^ To obviate at the unavailability of ROSE, a “Macroscopic on-site quality evaluation” (MOSE) of the samples is suggested to estimate the presence of tissue core; however data are still sparse.^7^ Ex vivo fluorescence confocal laser microscopy (FCM) is an optical technology for rapid microscopic digital imaging of fresh unfixed biological specimens without any slide preparation; this technique is known as instant digital pathology.^8^ FCM involves no damage or cell/tissue loss during the examination and the sample can subsequently be recovered and formalin-fixed and paraffin-embedded (FFPE) for permanent histology. FCM allows both cellular and architectural details to be visualized, similar to standard histological analysis of paraffin-embedded or frozen samples. With FCM, multimodal confocal microscopy using different laser sources and fusion images can generate optical plans similar to those from hematoxylin/eosin (H&E)-stained sections directly from native tissues.^8^ FCM is rapidly emerging for real-time microscopic assessment of resection margins in various surgical fields including for skin tumors, urological neoplasms, and prostate cancer^9^^,^^10^; and for immediate diagnosis in different organs such as colon, prostate, and thyroid.^11^, ^12^, ^13^, ^14^ FCM appears particularly appealing for rapid bedside evaluation of small specimens, such as image-guided fine-needle aspiration biopsy, core needle biopsy, and endoscopic biopsy.^15^^,^^16^ Recently, a method to subject pancreatic EUS-FNA/FNB samples to FCM evaluation was reported based on a polymeric scaffold (Cytomatrix, UCS Diagnostic, Morlupo, Italy) as a holder to keep specimens under the FCM objective and maintain cellular material and fragments during subsequent FFPE preparation.^17^ This previous single-center study demonstrated good concordance between the FCM image-based evaluation and the final FFPE histology, supporting EUS-FNA/FNB's potential for improving the diagnostic workflow for solid pancreatic lesions. However, since FCM produces digital images via a new modality, pathologists may need to follow a learning curve to use it effectively. Thus, we performed a multicenter study by sharing FCM images and paired FFPE microscopic digital slides from EUS-FNB of solid pancreatic lesions, to evaluate pathologists' interobserver agreement (IOA) and confidence in this digital approach.

This project primarily aimed to assess IOA in determining sample adequacy and recognizing morphological clues in FCM, compared with paired FFPE sections. Secondary aims were to evaluate the inter-test agreement in reporting the diagnostic category and conclusive diagnosis in FCM and paired FFPE and to observe the variation in IOA among pathologists when evaluating digital FCM images and conventional FFPE.

## Methods

### Study population

EUS-FNB samples from solid pancreatic lesions of 25 consecutive cases were randomly extracted “in batch” from a database of over 140 specimens observed from April 2020 to May 2021 at the Pathology Unit of Campus Bio-Medico, Rome. All patients presented a pancreatic focal lesion and were referred to the Endoscopy Department of Campus Bio-Medico Hospital, Rome, to undergo EUS-FNB for microhistological and cytological diagnosis. For all procedures, an oblique-viewing linear endoscope (EG-580UT; Fujifilm, Tokyo, Japan) was used. A needle with a three-plane symmetric cutting surface, Franseen geometry designed for FNB (Acquire; Boston Scientific Corporation, Natick, MA, USA), was used for all examinations. The needle size (22 or 25 gauge) was chosen after considering the lesion size and location.

**Table:** Features of study population.

Sex	Age (Years)	Localization of the lesion	Diameter (mm)
**Male** 14	24–79	**Head** 16	14–53
		**Body** 5	
**Female** 11		**Tail** 3	
		**Uncinate process** 1	

### Tissue preparation

**Fig. 1:**
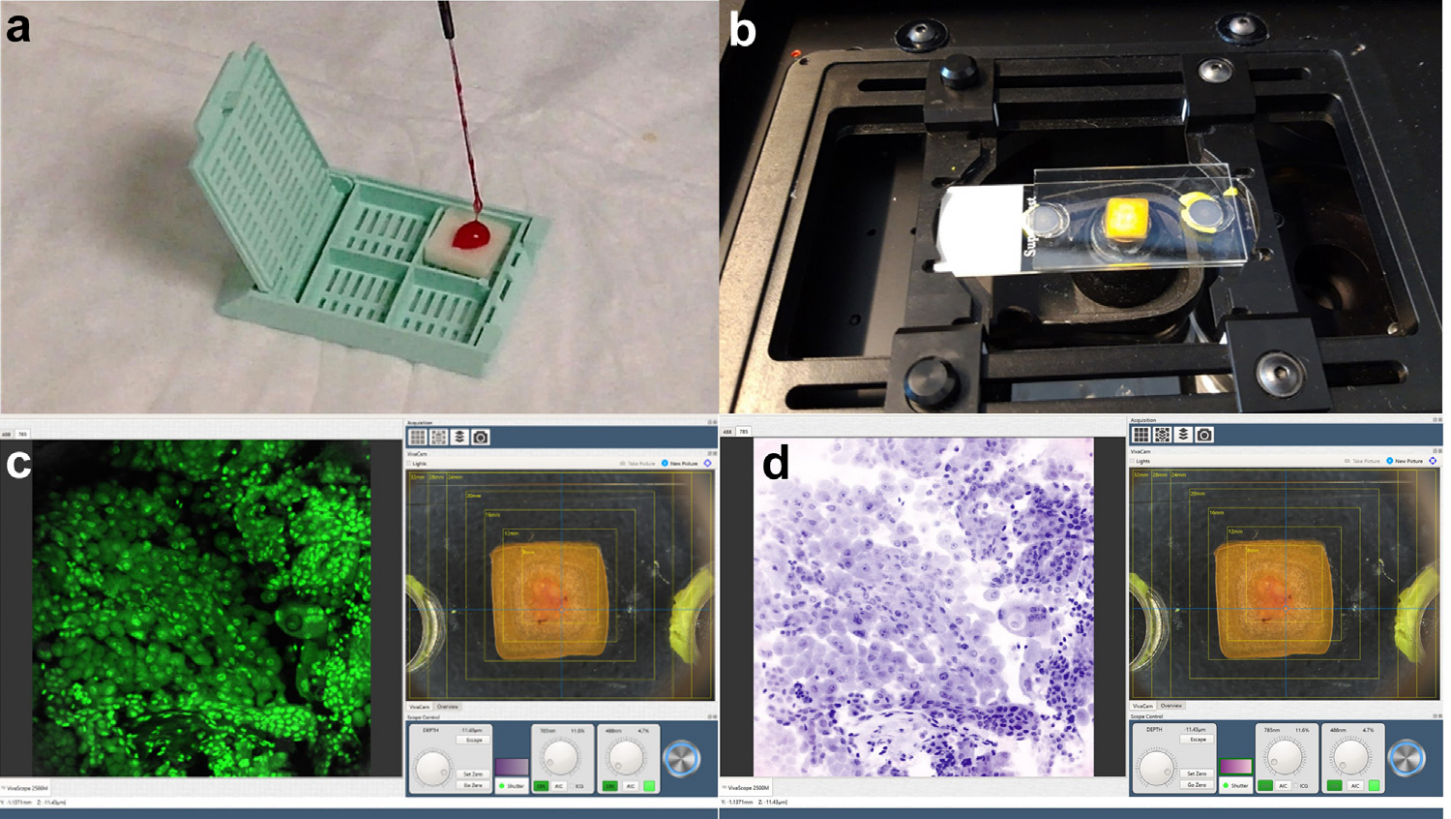
Sample preparation and imaging. Four steps of the process are showed. a. The sample is delivered on the polymeric scaffold immediately after EUS-FNB. b. The scaffold is put on a FCM dedicated microscopic slide and covered with a second slide in a sandwich manner, before placing it in the imaging device. c. Vivascope modality uses acridine fluorescence and the inherent light-reflection properties of tissue to generate optical sections. d. Vivascope software converts the reflectance and fluorescence greyscale mosaics into H&E pseudo-colored image.

After FNB, the needle was removed from the EUS endoscope, and the obtained specimen was entirely expelled and placed directly in a dedicated scaffold (Fig. 1a) (Cytomatrix; UCS Diagnostics). Cytomatrix is a CE-IVD diagnostic tool intended to produce a cell block directly from the needle avoiding needle washing and the following laboratory preparations. Cytomatrix consists of a naturally derived, foam-like porous substrate with positive charge on its surface that helps retain the cytological and microhistological material obtained by FNA/FNB sampling. The FCM VivaScope® 2500 (2500M-G4; VivaScope, Munich, Germany) was used for immediate imaging. For the Vivascope analysis, the Cytomatrix with FNB sample was used in its fresh state immediately after the loading: it was dropped with buffered saline solution and the excess fluid was drained away thanks to the porous structure of the matrix. This step was followed by dropping acridine-orange solution for 20 s on the sample and a rapid washing with buffered saline. Then, Cytomatrix was put on a FCM dedicated microscopic slide and covered with a second slide in a sandwich manner, before placing it in the imaging device (Fig. 1b). The Vivascope device has a vertical resolution of 4 micron and a maximum examination depth of 200 micron; the maximum total scan area is 25 × 25 mm. This microscope is equipped with a 38% water immersion objective with a numerical aperture of 0.85 (VivaScope - Technical data, 2019, available at: https://www.vivascope.de/wp-content/uploads/2019/06/DS_VS-2500M-G4_287_0219-ohne-Mohs.pdf). The digital staining modality was used to convert the reflectance and fluorescence grayscale mosaics into H&E pseudocolored images (Fig. 1c and d). Cytomatrix is suitable for formalin fixation followed by paraffin embedding, so, after FCM examination, the scaffolds were recovered from the Vivascope slot, and, in order to obtain a permanent cellblock, they were formalin fixed and paraffin embedded after alcohol dehydration and xylene clearing as for routine histology. Four-micron-thick sections were cut from the paraffin blocks and stained with H&E.

### Study design

This multicenter study was based on digital sharing of previously collected microscopic images from pancreatic EUS-FNB. Digital images from 25 consecutive cases, obtained with the VivaScope® GmbH 2500M-G4, and 25 paired whole-slide images (WSIs) from FFPE slides digitalized using Pannoramic 250 Flash III scan by Epredia (https://epredia.com/digital-pathology-solutions/) were shared among pathologists from 10 different international centers in Europe, Japan, and the United States. The participants specialized in pancreatic diseases, even if not trained in FCM. All pathologists were given controlled access to a slide center (SlidesCenter™ by Epredia) and received a table with clinical and sonographic baseline features for each case. A prior web meeting was held to explain how the VivaScope works and how the digital images are produced by transforming fluorescence and reflectance, induced by the lasers, in H&E pseudocolors. A web presentation and tutorial were shared by Epredia to explain how to access the SlidesCenter™ and how the system works. Technical support was available throughout the project. The study was planned in two rounds: one based on FCM digital image evaluation and the other on evaluating digital slides of paired FFPE sections, with a random slide order in a blinded manner. A questionnaire structured in four levels (questions: Q) was implemented. In each round, pathologists were asked to report on sample adequacy (Q1) and, when adequate, to categorize the 25 cases (Q2) as negative, atypical/suspicious of malignancy, or neoplastic/malignant. They were also asked to complete a table reporting the presence/absence of 10 morphological clues for each sample (Q3) and finally to report a diagnostic hypothesis (Q4). After completion of a round, tables and images remained unavailable for consultation. A week's washout interval was set between the first and second rounds. Both datasets, from VivaScope interpretation and FFPE digital slide evaluation, were collected on a web-based table and answers were summarized in Excel files for statistical analysis. The complete study design is summarized in the following diagram and detailed in Supplementary Material 1.

**Figure:**
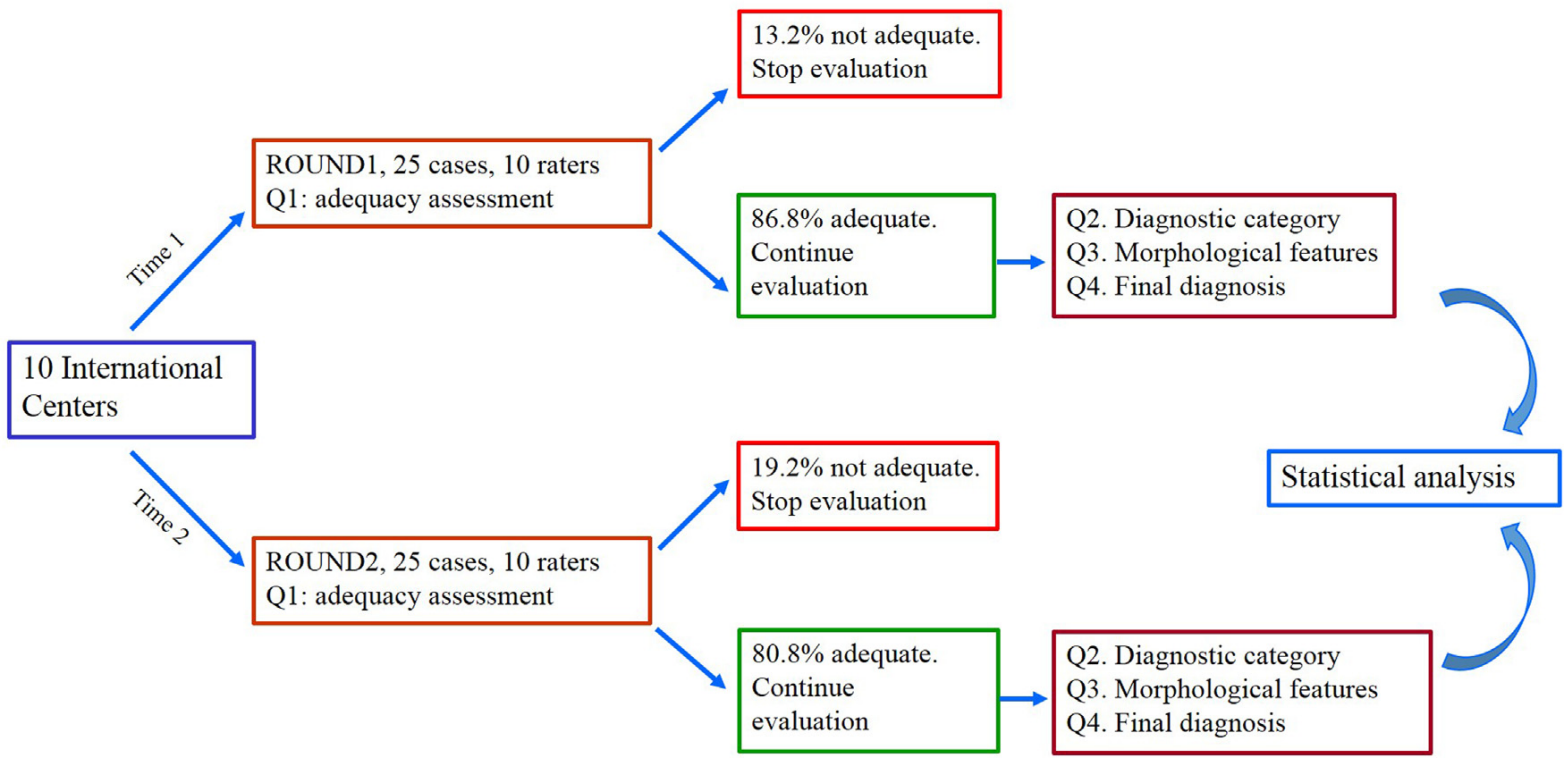

### Statistical analysis

Each patient's sample (n = 25) underwent the two different imaging procedures (FCM and FFPE) for a total of 50 images. Each image was then assessed by 10 different pathologists who judged the images independently from each other and were blinded to the pairing (which two images belonged to the same patient was obscured). Our final analytical sample therefore included 500 observations.

We considered various agreement coefficients to compare FCM and FFPE, using methods and formulas discussed by Gwet (2014),^18^ namely, percent agreement, Cohen's kappa, Gwet's AC1, the Brennan and Prediger coefficient, Fleiss's kappa, and Krippendorff's alpha coefficient.

We adopted two statistical approaches. First, we carried out a paired inter-test comparison between the FCM and FFPE images (250 vs. 250 observations for each comparison). The coefficients’ 95% CI was calculated using bootstrapped standard errors (1000 replications) to account for the clustering by rater (pathologist). Second, we evaluated the agreement among the 10 raters in judging each image (inter-rater agreement) and then compared the results between ex vivo and FFPE using unpaired t-tests (25 vs. 25 observations for each comparison).

While Cohen's kappa is widely used for assessing inter-rater reliability, it has well-documented statistical problems, especially when the number of raters exceeds two.^19^ Gwet's AC1 was shown to overcome these limitations and provide a more stable inter-rater reliability coefficient.^20^^,^^21^ We thus report only Gwet's AC1 coefficients, while the other coefficients are shown in the appendix for the reader's benefit. Gwet's AC1 coefficients can be interpreted using the same benchmarks as the Kappa statistics (i.e., values ≤ 0.20 indicate poor agreement, 0.21–0.40 fair, 0.41–0.60 moderate, 0.61–0.80 good, and 0.81–1.00 very good agreement).

This study aimed to evaluate the interobserver agreement among pathologists using two different diagnostic technologies, Vivascope and FFPE, with no reference diagnosis. To assess the cancer prevalence in the cases, we used a consensus-based method to define the gold standard for diagnosing pancreatic cancer: malignancy was confirmed if at least five out of ten raters reported malignancy at the FFPE assessment.

### Ethics

This study conforms to the ethical guidelines of the 1975 Declaration of Helsinki and was reviewed and approved by the local Institutional Review Board of Campus Bio-Medico University of Rome (Prot.: 47.2(20)0.21 ComEt CBM). Written informed consent was obtained from all patients before the endoscopic examination procedures.

### Role of funders

This study was not supported by any funding source.

## Results

The study ran from June to July 2021 and was successfully completed. All Centers completed the survey. Complete statistical data are available in Supplementary Material 2.

With regard to the agreement between the two tests, for Q1, the coefficient was 0.79 (95% CI 0.65–0.92) when the parameter was used as a binary variable (adequate vs. inadequate sample). Moreover, the coefficient was 0.80 (95% CI 0.70–0.89) when Q1 was considered as a categorical variable with five possible answers. For Q2, the coefficient of agreement was 0.67 (95% CI 0.54–0.81) when the parameter was used as a categorical variable with three possible answers and 0.66 (95% CI 0.50–0.83) when binary (malignant vs. benign/atypical). In addition, there was no evidence of a difference between FCM and FFPE in terms of inter-rater agreement when the question was treated as a binary variable (0.58 vs. 0.87, P = 0.115 Gwet), but there was some evidence of a difference when it was treated as a categorical variable, with higher agreement in Round 2 (0.64 vs. 0.89, P = 0.076).

Table 1 shows the inter-test agreement coefficients from all of the questions. Table 2 shows the agreement coefficients among the ten raters for each question and for each imaging technique separately. There was no evidence of a difference between ex vivo and FFPE in terms of inter-rater agreement for Q1, as the coefficient was 0.81 in Round 1 (FCM) and 0.73 in Round 2 (FFPE) when the question was treated as a binary variable (adequate vs. inadequate, P = 0.427 Gwet) and 0.82 vs. 0.73 (P = 0.290 Gwet) when it was treated as a categorical variable.

**Table 1:** Paired inter-test agreement (2 tests, 25 patients, 10 raters) N = 250.

Question	Agreement coefficient (Gwet)	(Bootstrapped 95%CI)
Q1 bin	0,79	(0,65–0,92)
Q1 cat	0,80	(0,70–0,89)
Q2 bin	0,66	(0,50–0,83)
Q2 cat	0,67	(0,54–0,81)
Q3A	0,45	(0,29–0,60)
Q3B	0,82	(0,77–0,86)
Q3C	0,70	(0,56–0,83)
Q3D	0,35	(0,17–0,54)
Q3E	0,61	(0,42–0,79)
Q3F	0,32	(0,16–0,48)
Q3G	0,25	(0,08–0,42)
Q3H	0,61	(0,52–0,71)
Q3I	0,55	(0,44–0,66)
Q3L	0,37	(0,15–0,60)
Q4	0,43	(0,28–0,58)

**Table 2:** Inter-rater agreement (10 raters).

Question	Agreement coefficient (Gwet) with 95%CI				P(Gwet)
	ROUND 1		ROUND 2		
Q1 bin	0,81	0,67–0,94	0,73	0,58–0,87	0,427
Q1 cat	0,82	0,71–0,93	0,73	0,60–0,86	0,290
Q2 bin	0,58	0,31–0,85	0,87	0,67–1,00	0,115
Q2 cat	0,64	0,44–0,84	0,89	0,73–1,00	0,076
Q3A	0,36	0,19–0,54	0,52	0,27–0,77	0,251
Q3B	0,80	0,65–0,95	0,96	0,85–1,00	0,120
Q3C	0,47	0,28–0,65	0,72	0,55–0,89	0,053
Q3D	0,43	0,24–0,63	0,26	0,06–0,46	0,222
Q3E	0,58	0,39–0,77	0,93	0,83–1,00	0,009
Q3F	0,31	0,12–0,51	0,30	0,11–0,49	0,927
Q3G	0,04	−0,06–0,13	0,18	−0,02–0,38	0,110
Q3H	0,59	0,43–0,74	0,67	0,46–0,87	0,499
Q3I	0,42	0,23–0,61	0,57	0,32–0,81	0,306
Q3L	0,17	0,03–0,31	0,29	0,20–0,39	0,178
Q4	0,43	0,28–0,58	0,52	0,33–0,71	0,420

Using the Qwet's AC1 test, there was no evidence of a difference between FCM and FFPE for Q3A (0.36 vs. 0.52, P = 0.251), or for Q3B (0.80 vs. 0.96, P = 0.120), Q3D (0.43 vs. 0.26, P = 0.222), Q3F (0.31 vs. 0.30, P = 0.927), Q3G (0.04 vs. 0.18, P = 0.110), Q3H (0.59 vs. 0.67, P = 0.499), Q3I (0.42 vs. 0.57, P = 0.306), Q3L (0.17 vs. 0.29, P = 0.178), and Q4 (0.43 vs. 0.52, P = 0.420). However, for Q3C, the agreement was higher in Round 2 (0.47 vs. 0.72, P = 0.053), as for Q3E (0.58 vs. 0.93, P = 0.009).

Using the consensus-based method, cancer prevalence in our study was 68% (17/25), and the positive predictive value of VivaScope was 100%, since malignancy was reported for the same 17/25 cases on both VivaScope and FFPE images.

## Discussion

Digital pathology is a constantly evolving field. Herein, we used IOA among pathologists to investigate the value of modern digital FCM for immediately assessing histo-cytological samples of pancreatic masses compared with IOA using conventional digital pathology. In particular, IOA was evaluated for real-time assessment of adequacy and morphology directly on native unfixed specimens. The substantial interest in EUS-FNB-based diagnosis of pancreatic lesions is largely demonstrated by wide investment in new sampling devices and in modern needles able to obtain cytological material and tissue cores simultaneously.^22^ The weak link in this workflow is the lack of significant innovation to manage sampled material and to speed up its microscopic analysis. Among FCM instruments, the VivaScope modality can generate optical sections of biological specimens by the conversion of laser signals into a very realistic simulation of H&E staining. This ability shortens the learning curve and opens the way for use in routine pathology practice.

Examination of specimens from EUS-sampled solid pancreatic lesions is difficult for pathologists, showing moderate IOA for final diagnosis and low to fair IOA for individual cytological parameters using conventional microscopy.^23^ Our study is focused on IOA using CFM for evaluating the adequacy and assignment of a diagnostic category to specimens obtained during EUS-FNB for pancreatic solid masses.

The first endpoint was the interobserver agreement in assessing sample adequacy using the FCM technology. Using Gwet's AC test, IOA was very good for FCM images and good for FFPE WSIs, albeit without a significant difference. Adequacy was reported in 419/500 observations (83.8%). Considering that FCM images are obtained within 4 min including the acridine procedure, scanning, and image display, and that fresh samples remain intact during analysis, this technique is clearly advantageous. Moreover, the confocal modality allows the whole sample to be observed by moving along the X, Y, and Z axes, enabling the quantification of pathological cells to predict the adequacy for diagnosis as well as for molecular analysis. The concordance in adequacy between VivaScope images and paired FFPE sections was good (Gwet's AC 0.79, 95% CI 0.69–0.92), confirming the availability of adequate specimens while avoiding cell smearing as in ROSE or compensating for the lack of ROSE availability. Additionally, FCM extends beyond immediate adequacy assessment. On the same FCM images, pathologists can classify cases into reporting categories as suggested for the diagnosis of pancreatic lesions on permanent cytological samples.^24^ The real-time assessment appeared reliable with an immediate impact on patient management. Considering the observations of single pathologists, the IOA for diagnostic categories between VivaScope images and permanent FFPE sections was moderate (0.56, 95% CI 0.44–0.69) and there was no significant difference in IOA when using the two sets (0.48 for FCM, 0.56 for FFPE; Gwet P = 0.462). Among “atypical/suspicious” answers on FCM (42/250 answers), 20 (47.6%) received a final assignation of “malignant” on the FFPE slides. This may be the result of low confidence among pathologists in VivaScope images due to it being their first use of instant pathology, leading to cautious interpretation. Meanwhile, 144/250 answers were “malignant” on VivaScope images, with 119 (82.64%) being confirmed as malignant on permanent sections, supporting good intra-observer reproducibility regarding the immediate information about the presence of cancer.

**Fig. 2:**
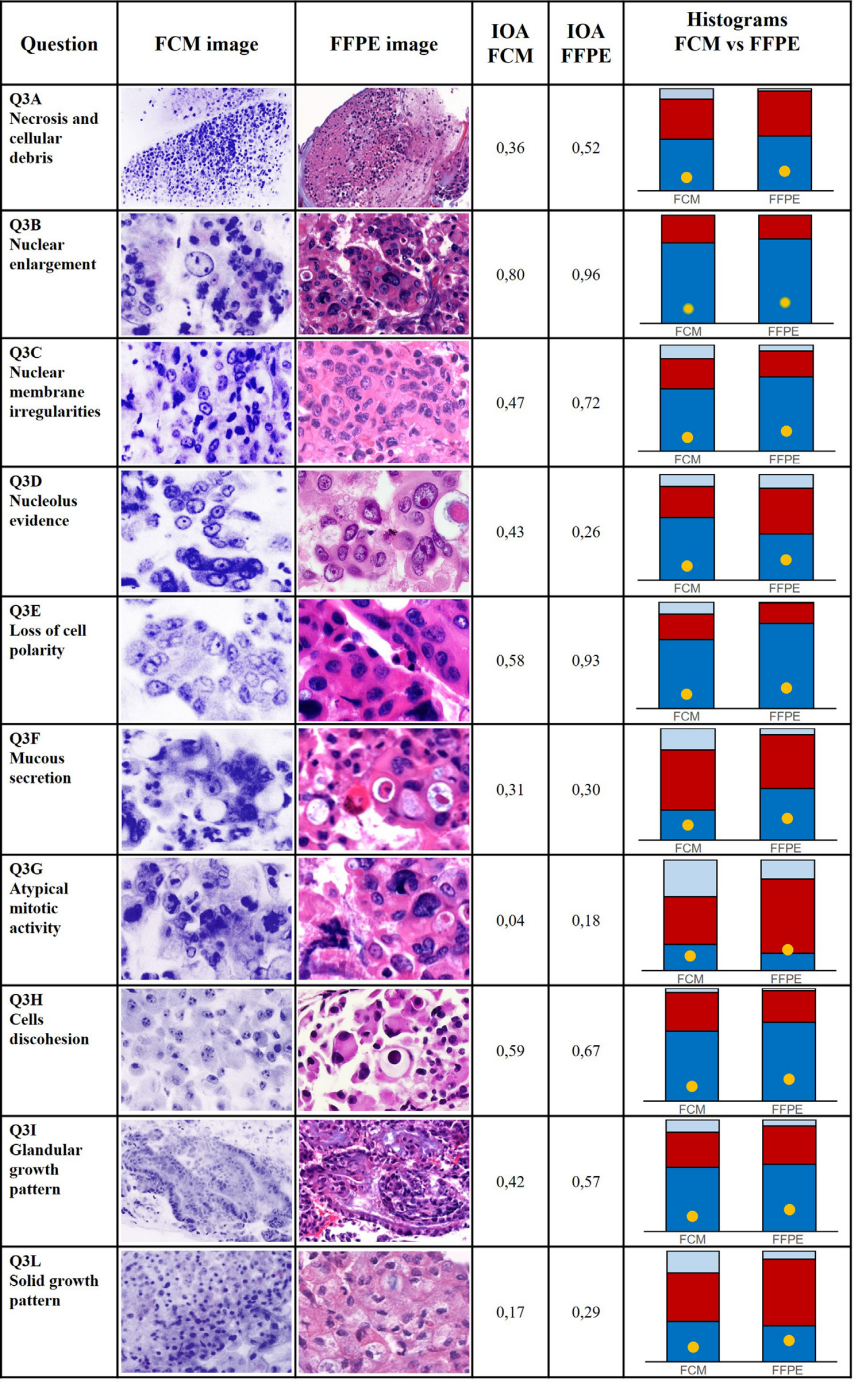
Diagnostic clues evaluation. Detailed representation of Q3 about diagnostic clues. In the first column the question is right-hand, in the second and in the third are shown images of the morphological detail in FCM and FFPE mode respectively, in the fourth and in the fifth column the respective coefficients of IOA are reported, and in the last column the relative histograms. In the histograms. Blue: present; Red: absent; gray: not evaluable. Yellow dot: sample not evaluated due inadequate designation in Q1.

Q3 contributed to elucidating the role of specific morphological details (Fig. 2). Among the 10 key morphological features analyzed by pathologists in diagnosing pancreatic tumors, nuclear enlargement was the most consistent clue with good IOA on FCM (0.80), very good IOA on permanent FFPE (Gwet 0.96, P = 0.120), and very good inter-test agreement (0.82). Neoplastic architecture (solid or glandular growth pattern) was recognized with fair-to-moderate agreement, possibly for intrinsic features of these samples that are a mixture of cellular material and core of tissue. Nucleolar prominence was best enhanced in FCM images (IOA 0.43), followed by FFPE sections (IOA 0.26) probably due to nucleolar avidity for acridine orange dye. Necrosis and lack of cellular cohesion resulted in consistent diagnostic clues. There were no significant differences in IOA in their evaluation on FCM and FFPE sections and there was moderate and good concordance, respectively, between the two techniques. Nuclear membrane irregularities were best assessed on permanent FFPE (0.72), followed by FCM (Gwet 0.47; P = 0.038). This is expected since acridine orange does not react with cell or nuclear membranes and the nuclear border is only evidenced on FCM by chromatin margination. The most difficult evaluation was the presence of atypical mitotic activity, with poor agreement among pathologists using both FCM and FFPE images. Loss of cell polarity appears to be less easy to recognize on FCM, with moderate IOA on FCM (0.58) and very good IOA on FFPE (Gwet 0.93; P = 0.009). Mucous secretion remains a challenging feature to assess. A previous study demonstrated fair interobserver agreement for grading mucinous neoplasms and low interobserver agreement for identifying neoplastic mucin in conventional samples from EUS pancreatic needle aspiration.^25^ Our study confirms this difficulty, showing fair interobserver agreement for both FCM images (0.31) and final FFPE (0.30), without a significant difference (Gwet P = 0.927). The finding of interobserver agreement among pathologists in assessing detailed morphological features, regardless of the technology used, is valuable and could aid in the standardization of histological evaluation.

Finally, our data suggest that VivaScope images may be suitable for immediate definitive diagnosis. We obtained moderate interobserver agreement on both FCM (0.43) and FFPE (Gwet 0.52; P = 0.420). Ductal adenocarcinoma is the main cause of pancreatic masses, and this diagnosis was reported on VivaScope images in 92/250 observations and confirmed on permanent FFPE in 70.8%. Notably, when considering cases as positive when at least five raters report them as malignant, the positive predictive value of VivaScope was 100%. Other neoplastic conditions can be detected in the pancreas and distinguishing different types of pancreatic mass is an important clinical challenge for establishing the optimal treatment. In these cases, an integrative analysis using immunohistochemical or molecular testing may be necessary. Overall, 42/419 observations (10.0%) were reported as “neoplasms to be characterized by further analyses.” The availability of a cell block from FCM-analyzed material allows any ancillary assessment and final exhaustive diagnostic reporting. However, diagnosis of benign pancreatic pathology remains a challenge: Benignity was diagnosed in 30/250 answers with VivaScope, 13 of which were confirmed as benign on paired FFPE sections (43.3%). This reflects an already-encountered difficulty for conventional diagnoses on EUS-FNB of solid pancreatic lesions. In a meta-analysis of 828 patients undergoing EUS-FNB for pancreatic masses, the negative likelihood ratio (NLR) was found to be 0.17, suggesting that EUS-FNB is valuable for a rule-in diagnosis, while NLR was not sufficiently low to rule out a malignant diagnosis.^5^

This study has some potential limitations. First, all analyses were performed on digital platforms. Although this is a modern trend in pathology, the use of digital cyto-histological images is not yet a routine practice in diagnostics. The equivalence between FCM and digital FFPE images is supported by the finding that IOA was generally similar between the two systems used. Overall, the results of this study suggest that instant digital pathology using FCM technology requires a learning curve similar to that required for managing conventional digital microscopy. It may improve with routine use and comparison with paired conventional paraffin sections to increase the familiarity and confidence of pathologists in using these systems over time. Second, the study focused on immediate assessment, so, at the time of the evaluation, integrative analysis using immunohistochemical or molecular testing that may be necessary to differentiate tumor histotypes was not available. Finally, a small proportion of “malignant” answers from FCM images were downgraded to lower risk classes for FFPE (14/144, 9.72%). This finding may be regarded as either a potential overestimation on FCM, requiring a longer learning curve, or a better opportunity of assessment on FCM images.

This study was directed to the evaluation of IOA in actual clinical practice, so for all cases clinical and sonographic information was provided when pathologists evaluated the images. Such clinical information is usually available at the time of on-site evaluation and facilitates specimen appraisal. Previous studies reported that, in remote consultation of digital microscopic images for diagnostic purposes, data from a clinical setting and other downstream processes (i.e., laboratory information) should be included in concordance studies.^26^ Moreover, clinical, imaging, and laboratory information is required when using diagnostic reporting systems.^27^

FCM images can be evaluated remotely in real time without the need for the pathologist to be on site or can be analyzed later for a second post-processing consultation. These characteristics, typical of digital pathology, make FCM a tool that can be perfectly integrated into digital workflows of histo-cytological diagnostics. In an educational setting, VivaScope software allows the annotation of digital images in order to create digital atlases/collections to offer tutorials in digital pathology in the puzzling diagnostics of pancreatic EUS-biopsies.

A final consideration focuses on the application of artificial intelligence algorithms to FCM digital images, overcoming current limits of conventional digital histology. Acridine orange emission channel alone produces grayscale images similar to radiological diagnostics, for which a consolidated application of interpretative algorithms already exists. Moreover, using the ability of the device to eliminate the “out-of-focus” brightness at a range of magnifications, a feature typical of FCM,^28^ the thickness of the optical planes obtained by VivaScope is regularly 4 microns, avoiding the thickness variations of conventional paraffin sections. Supervised deep learning algorithms are a hot topic in digital pathology and may integrated into a future standard of care.

### Conclusion

This leading multicenter study on the use of FCM for the diagnostic interpretation of pancreatic EUS/FNB confirms the role of instant digital tools as valid alternative to traditional microscopy. In particular, FCM may improve adequacy assessment and real-time diagnostics in solid lesions, thereby reducing unnecessary delays in patient management, with significant implications for planning modern diagnostic workflows for pancreatic tumors.

## Contributors

AC and FdM had the original idea, wrote the study protocol, coordinated the study procedures, and critically revised the report. AC, CT, SS, and MV collected cases, prepared digital images and monitored the progress of the study. AC, MV and SS prepared the web tables. JB, ArC, MC, CD, NF, ZL, JL, AP, FP, and LRB performed the evaluation of digital images from the fifty cases and reported their observation in the web tables. IA, FF, GL cared for data collection. AC and MV supervised the statistical analysis. MV performed the graphical representation. FdM, PGA, AG, and MG contributed to data interpretation. AC, MV and CT wrote the first draft of the paper. The chief investigator (AC), and SS, FF, and FdM had full access to all data, once the database was locked and verified underlying data. All authors revised and approved the final version of the manuscript. AC and FdM were responsible for the decision to submit the manuscript.

## Data sharing statement

We will consider sharing de-identified, individual participant-level data that underlie the results reported in this Article on receipt of a request detailing the study hypothesis and statistical analysis plan. All requests should be sent to the corresponding author. The corresponding author and lead investigators of this study will discuss all requests and make decisions about whether data sharing is appropriate based on the scientific rigour of the proposal. All applicants will be asked to sign a data access agreement.

## Declaration of interests

AC and CT are co-authors of a patent issued owned by 50% UCS Diagnostics srl (Italy) and 50% University Campus Bio-Medico (Rome, Italy). The other authors declare no competing interest.

## Appendix A Supplementary data

Supplementary material 1

Supplementary material 2A

Supplementary material 2B

Supplementary material 2C
