# Supplementary material for: New digital confocal laser microscopy may boost real-time evaluation of endoscopic ultrasound-guided fine-needle biopsy (EUS-FNB) from solid pancreatic lesions: Data from an international multicenter study
Source: eBioMedicine. 2022 Nov 24;86:104377. doi: 10.1016/j.ebiom.2022.104377 (PMC9706538; doi:10.1016/j.ebiom.2022.104377)
Supplement: Supplementary material 1 [file mmc1.docx]

**Supplementary material 1 Complete study design**

**Starting modalities:** We extracted 25 consecutive cases from a database of over 140 specimens observed from April 2020 to May 2021 at the Pathology Unit of Campus Bio-Medico, Rome. The first number was randomly selected by computer; this case and the following 24 cases were collected for the study. The 25 digital images from 25 patients were acquired using the Vivascope software, including the macro-image of the loaded scaffold and the micro-image of the sample; 25 physical slides of the paired permanent FFPE sections were digitalized using Pannoramic 250 Flash III scan by Epredia. Image~~s~~ storage and sharing was supported by SlideCenter^TM^. SlideCenter^TM^ is a powerful, server-based slide management system with a fully featured slide database capable of storing both macroscopic images and digital slides. Thanks to its flexible structure, it can be adapted to several fields, including various research applications and medical education. SlideCenter^TM^ is developed by 3DHistech and is exclusively distributed in selected Countries, by Epredia (<https://epredia.com/digital-pathology-solution>s/). The study involved pathologists from 10 different international centers in Europe, Japan, and the United States.

Map of participating Centers:


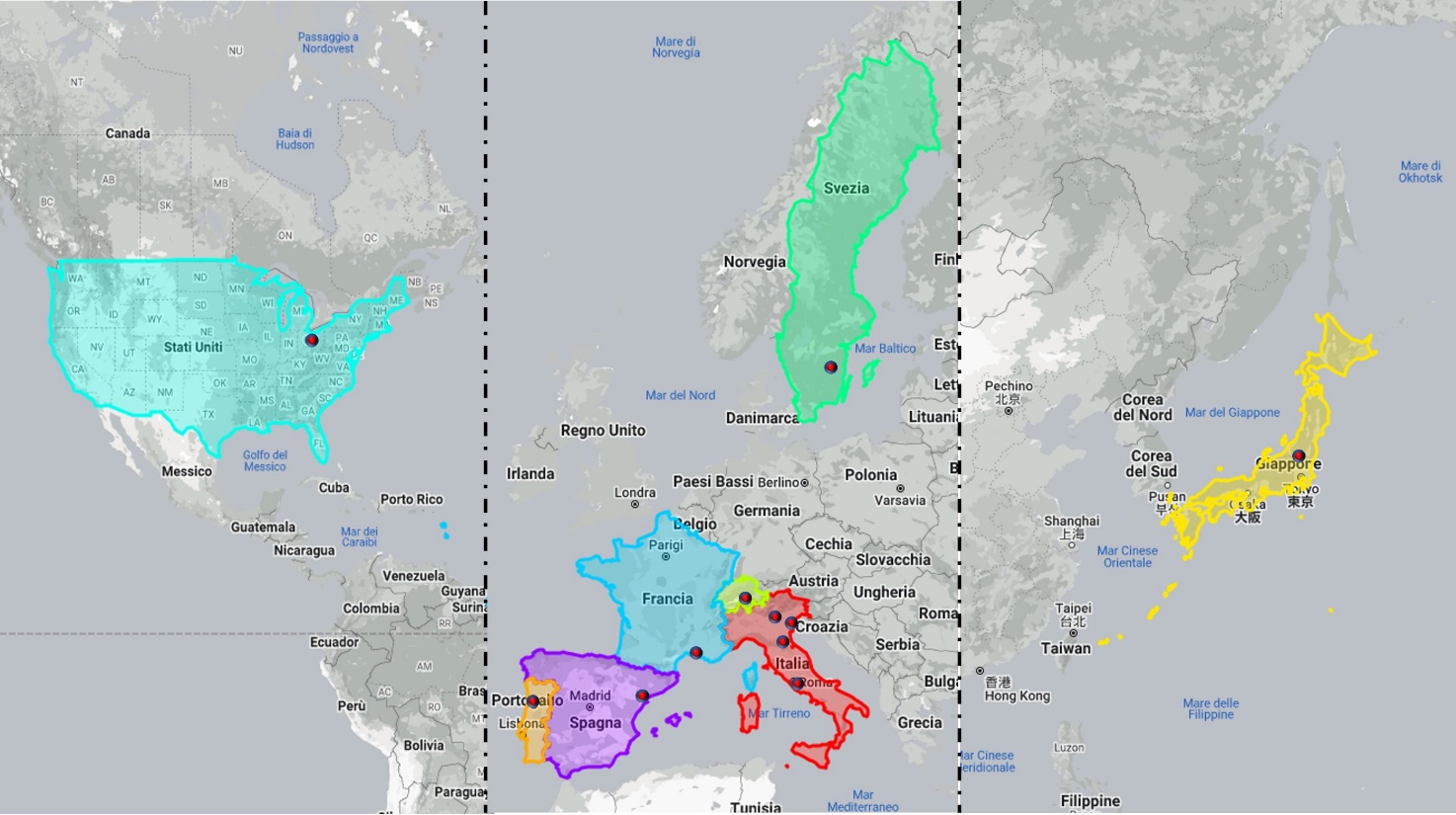


To access to the system, participating Centers received their own account and they could log in by Username and Password. The study was planned for the participation of 10 Centers. After entering in the SlideCenter^TM^, it was possible to visualize the images by the Case Viewer^TM^ and using the mouse, it was possible to move and navigate the images and to zoom them up to 500x.

A web-meeting was held to explain how the Vivascope works and how the digital images are produced by transforming fluorescence and reflectance, induced by the lasers, in false hematoylin/eosin colors. A web-presentation and tutorial was shared by Epredia, to explain how to access to the SlidesCenterTM and how the system works. Technical support was available throughout the project time.

**ROUND 1,** Time lapse: 20 days

After entering the system, the participant had access to digital images both from macroscopic and microscopic views obtained with VivaScope® 2500M-G4 from fresh unfixed samples from 25 cases of EUS- FNB of solid pancreatic lesions. Cases were numbered from 1 to 25. Each participant also received a link to open the web-based table with corresponding clinical and sonographic baseline features and the survey for the observed images. The survey was structured in four levels (Q):

**Q1. Adequacy of the sample** with five options (only one possible choice):

Inadequate because not representative of the lesion; Inadequate for technical artifacts, Inadequate for paucity of cells, Inadequate for blood contamination, Adequate for interpretation. Diagnostic adequacy is defined as procurement of a sample that was sufficient for cytological/histological interpretation: i.e. specimen contains evaluable groups of cells, not obscured by blood or damaged by sampling or technical artifacts (*Kong F, Zhu J, Kong X, Sun T, Deng X, Du Y, Li Z. Rapid On-Site Evaluation Does Not Improve Endoscopic Ultrasound-Guided Fine Needle Aspiration Adequacy in Pancreatic Masses: A Meta-Analysis and Systematic Review. PLoS One. 2016 Sep 22;11(9):e0163056. doi: 10.1371/journal.pone.0163056. PMID: 27657529; PMCID: PMC5033338*).

It was possible to proceed with the questioner only if “Adequate for interpretation” is checked.

**Q2. Diagnostic group if adequate** (based on The Papanicolaou Society of Cytopathology System for Reporting Pancreaticobiliary Cytology (*Martha Bishop Pitman, Lester James Layfield. The Papanicolaou Society of Cytopathology System for Reporting Pancreaticobiliary Cytology, Springer Cham, 2015; DOI* [*https://doi.org/10.1007/978-3-319-16589-9*](https://doi.org/10.1007/978-3-319-16589-9)), (only one possible choice):

1) Benign/Negative for malignancy (equivalent to Category 2 The Papanicolaou Society of Cytopathology System for Reporting Pancreaticobiliary Cytology);

2) Atypical/Suspicious (equivalent to Category 3 and 5 The Papanicolaou Society of Cytopathology System for Reporting Pancreato-biliary Cytology);

3) Neoplastic/Malignant (equivalent to Category 4 and 6 The Papanicolaou Society of Cytopathology System for Reporting Pancreato-biliary Cytology).

**Q3. Diagnostic features** with ten morphological details to be evaluated **as present, absent or not evaluable:** Necrosis, cellular debris; Nuclear enlargement; Nuclear membrane irregularities; Nucleolar evidence; Loss of cells polarity; Mucous secretion; Atypical mitotic activity; Cell discohesion; Glandular growth pattern; Solid growth pattern.

Finally, the pathologists were asked to make a diagnostic hypothesis possibly on the sole morphological ground or, alternatively, to indicate the need for integrative analyses such as immunohistochemistry.

**Q4. Cyto-histological Diagnosis:** Pancreatitis/atrophy; Serous cystadenoma; Intraductal papillary mucinous neoplasm/mucinous cystic neoplasm; Solid pseudopapillary neoplasm; Neuroendocrine tumor/carcinoma; Suspicious for carcinoma; Duct adenocarcinoma; Acinar cell carcinoma; Metastatic disease; Other (specify).

All the answers were automatically collected on an excel file for the statistical analysis.

At the end of Round 1 the images will not be longer available.

A week of washout interval was left between the two rounds.

**ROUND 2,** Time lapse: 20 days

After re-entering in the system, the participant got access to digital images obtained with Pannoramic 250 Flash III scan by Epredia from slides of formalin fixed paraffin embedded samples of the same scaffolds of the previous 25 cases of EUS -FNB of solid pancreatic lesions. Cases were listed in a different randomly settled order than the FCM case series and identified by letters A to Z. Each participant also received a link to open the web-based table with corresponding clinical and sonographic baseline features and the survey for the observed images. The survey was structured in the same four levels of details as for Round1: **Q1.** **Adequacy of the sample, Q2. Diagnostic group if adequate, Q3. Ten** **Diagnostic features,** and, **Q4. Cyto-histological Diagnosis.**

All the answers were automatically collected on an excel file for the statistical analysis.

At the end of Round 2 the images were no longer available.
