# Supplementary material for: New digital confocal laser microscopy may boost real-time evaluation of endoscopic ultrasound-guided fine-needle biopsy (EUS-FNB) from solid pancreatic lesions: Data from an international multicenter study
Source: eBioMedicine. 2022 Nov 24;86:104377. doi: 10.1016/j.ebiom.2022.104377 (PMC9706538; doi:10.1016/j.ebiom.2022.104377)
Supplement: Supplementary File S1 [file mmc5.docx]

EBIOM-D-22-02372

**Caption for supplementary material**

**Supplementary material 1, Complete study design**. This section describes the study workflow in detail and includes the starting modalities, the map of participating Centers, and a description of the activities planned for each of the two Rounds of the study.

**Supplementary material 2, Statistical Analysis.** This section includes three excel files related to the results of the statistical analysis. In particular, the supplementary material 2A reports the data relating to the InterRater agreement, the supplementary material 2B describes the InterTest agreement and the supplementary material 2C is an overall InterRater agreement for questions Q2-Q4 considering all the samples even if reported not adequate.
